# Hyperpigmentation as a Primary Symptom of Vitamin B12 Deficiency: A Case Report

**DOI:** 10.7759/cureus.29008

**Published:** 2022-09-10

**Authors:** Anzal Jangda, Diana Voloshyna, Krishna Ramesh, Anan Bseiso, Tanveer Ahamad Shaik, Saman Al Barznji, Muhammad Usama, Faraz Saleem, Muhammad Abu Zar Ghaffari

**Affiliations:** 1 Internal Medicine, Ziauddin University, Karachi, PAK; 2 School of Medicine, University of Michigan, Ann Arbor, USA; 3 Internal Medicine, Ramaiah Medical College, Bangaluru, IND; 4 College of Medicine, Al-Quds University, Jerusalem, PSE; 5 Cardiovascular Medicine, University of Louisville School of Medicine, Louisville, USA; 6 Internal Medicine, University of Sulaimani, Sulaymaniyah, IRQ; 7 Acute Medicine, University Hospitals of Derby and Burton, Burton, GBR; 8 Internal Medicine, Akhtar Saeed Medical and Dental College, Lahore, PAK

**Keywords:** cutaneous manifestation of vitamin b12 deficiency, adisonian mimick, vitamin b12 deficiency symptoms, cutaneous hyperpigmentation, hyperpigmentation

## Abstract

The presentation of vitamin B12 deficiency varies from being asymptomatic to affecting multiple organ systems. In addition, several systemic diseases can be associated with generalized weakness and hyperpigmentation. However, vitamin B12 deficiency rarely presents with hyperpigmentation as an initial symptom. We present a rare case of a 22-year-old college student who presented with hyperpigmentation as the only physical manifestation of early vitamin B12 deficiency. This case underlines the need to rule out vitamin B12 deficiency when clinicians encounter hyperpigmentation as a solo presentation and also emphasizes the significance of early treatment in preventing the irreversible neurological manifestations of vitamin B12 deficiency.

## Introduction

The deficiency of vitamin B12 can cause specific skin manifestations, such as hyperpigmentation, vitiligo, angular stomatitis, and hair and nail changes [1]. However, the most common dermatological manifestation is hyperpigmentation [2], which often occurs in combination with systemic findings, including macrocytic anemia, pancytopenia, and subacute combined degeneration of the cord (SCD) [3]. When dermatological features occur in the dissociation of systemic findings, the deficiency of vitamin B12 can be overlooked. As a result, such cases are rarely reported [1].

## Case presentation

**Figure 1:**
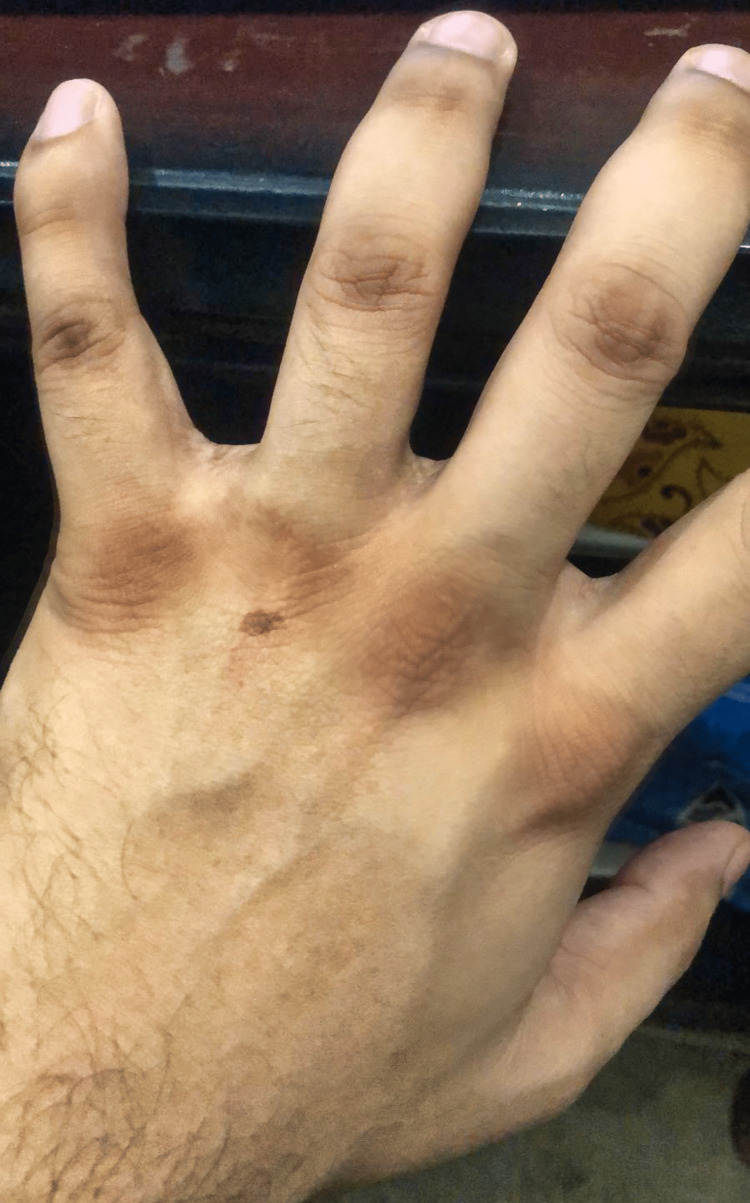
Hyperpigmentation of the left hand's knuckles and dorsal aspects of the interphalangeal joints.

A 22-year-old male student presented with a four-month history of the progressive development of hyperpigmentation on his knuckles. At first, he thought sun exposure was the primary cause, so he started wearing gloves during the day to prevent any exposure. Nevertheless, his hyperpigmentation gradually worsened with time, involving his knuckles, dorsal aspect of interphalangeal joints, distal phalanges, and the foot (dorsal surface of interphalangeal joints). His fingernails and the palmar surfaces of his hands and feet were spared. He denied the development of any rash or skin allergy before the onset of hyperpigmentation. Apart from hyperpigmentation being his only primary presentation, no other systemic complaints were present in the patient. His previous medical and medication history was unremarkable. He had never undergone any surgery and had never been on a restricted diet. His BMI was 20, and his weight had been stable over the past 12 months. On examination, vital signs were stable with no pallor. However, considerable hyperpigmentation in the form of brownish discoloration was noted, which was more marked on the dorsal aspect of the hands (Figure 1). There were no signs of hyperpigmentation in the oral cavity. A detailed systemic examination revealed no positive findings, including unremarkable neurological assessments. Based on the physical findings, the suspicion of autoimmune disorders was ruled out.

Investigations were carried out primarily to rule out the systemic causes of hyperpigmentation. Investigations included complete blood counts, serum electrolytes, 8 a.m. plasma cortisol levels, vitamin B12, and serum folic acid levels. His morning cortisol and serum electrolytes were within normal ranges, excluding adrenal insufficiency (Table 1).

**Table 1:** Complete blood count parameters and serum electrolytes. Hb: Hemoglobin; MCV: Mean corpuscular value; Na: Sodium; K: Potassium; CI: Chloride.

Lab parameters	Value (normal range)
Hb (g/dL)	13.6 (13.5-17.5)
MCV (fl)	96.8 (80-100)
WBC (X10^9^/l)	7.2 (4.5-11)
Platelets (X10^3^/ul)	300 (150-400)
Serum Vitamin B12 (pg/mL)	125 (210-911)
Serum Iron (μg/dL)	110 (67-175)
Serum Folic Acid (ng/mL)	6.8 (2-20)
Serum Cortisol 8 a.m (μg/dL)	13.8 (5-23)
Na (mEq/L)	141 (136-146)
K (mEq/L)	3.8 (3.5-5)
Cl (mmol/L)	96 (95-105)

**Figure 2:**
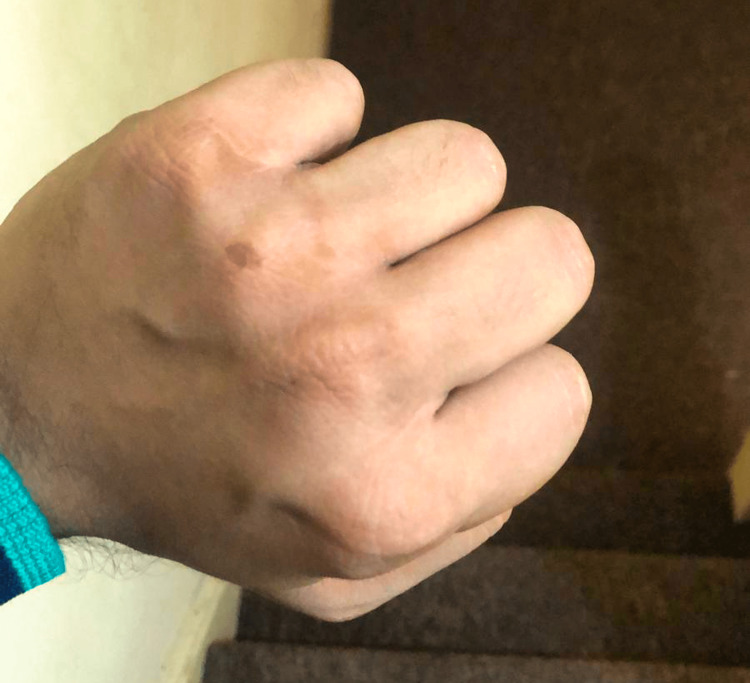
Post-treatment improvement.

His serum vitamin B12 levels were 125 pg/mL (210-911 pg/mL). All other investigations were normal. He was diagnosed with one of the rare early manifestations of vitamin B12 deficiency, as all other causes for hyperpigmentation were ruled out other than low serum vitamin B12 levels. Treatment with intramuscular injection of vitamin B12 was initiated (1000 mcg IM twice a week for a month, plus 100 mcg orally per day). Further investigations were carried out to rule out pernicious anemia. Moreover, improvement was noticed within two weeks of initiating treatment (Figure 2). 

At his second follow-up after three weeks, his vitamin B12 levels were over 300 pg/mL, and his hyperpigmentation was completely resolved. The antibody titer for pernicious anemia was also unremarkable, making a lack of vitamin B12 in the diet the sole cause of this occurrence. However, the oral dose of vitamin B12 of 100 mcg was continued for a month, and the patient was advised to increase their dietary intake of vitamin B12 from animal or fortified foods.

## Discussion

Vitamin B12 plays an integral role in the synthesis of DNA; its deficiency results in multisystem complications [2]. Plasma concentration below 200 pg/ml (148 pmol/L) is considered deficient. In the general population, the prevalence ranges from 3-5% to 5-20% among people aged >65 years [4]. The deficiency affects all age groups and causes a range of conditions [1], most commonly affecting the hematological and neurological systems [3]. The neurological complications include SCD, peripheral neuropathy, and psychiatric changes [2]. Many mucocutaneous findings, including hair and nail changes and hyperpigmentation, especially of the hands and feet, are associated with vitamin B12 deficiency [5].

Reversible skin and mucosal hyperpigmentation are the most commonly found skin manifestations of vitamin B12 deficiency [6]. However, most cases present alongside systemic findings, such as malabsorption, anemia, pancytopenia, and variable neuropsychiatric problems [3]. Interestingly Dr. Bramwell Cook defined a syndrome in late 1944 related to vitamin B12 deficiency, entailing hyperpigmentation, glossitis, and macrocytic megaloblastic anemia as its primary features. It has been observed that up to 1 in 5 patients with a deficient B12 level may have cutaneous hyperpigmentation [7]. Rarely, as observed in this patient, has skin hyperpigmentation been reported as the only symptom of vitamin B12 deficiency [4]. Other more common causes of hyperpigmentation include systemic causes, such as Addison disease, hyperthyroidism, hemochromatosis, and certain primary skin disorders. Protein-energy malnutrition, zinc deficiency, and pellagra can also cause hyperpigmentation [8].

In one study, Baker SJ et al. observed that 21 patients who presented with other primary findings of vitamin B12 deficiency also had hyperpigmentation [9]. Moreover, Aaron S et al. found that 41% (26 out of 63) of his patients presented with cutaneous changes as a primary symptom of vitamin B12 deficiency. While 52% of these 26 patients (22% of the total) presented with mucosal changes, glossitis was specifically seen in 31% (19 out of 63), hyperpigmentation of the skin in 19% (12 out of 63), hair changes in 9% (6 out of 63), angular stomatitis in 8% (5 out of 63), and lastly, vitiligo was seen in only 3% (2 out of 63) [10]. Therefore, regardless of the presence of neurological symptoms, SCD should be suspected in patients with skin hyperpigmentation, as the neurological manifestations can be a late finding [2].

Skin hyperpigmentation in B12 deficiency was most commonly observed on the hands and feet (dorsal aspect), with the knuckles being the most prominent site. The darkening of sole and palmar creases, interphalangeal joints, and terminal phalanges was also observed [3,4,5]. It remains questionable whether the development of hyperpigmentation is responsive to specific serum vitamin B12 levels [2].

The pathophysiologic mechanism of hyperpigmentation involves increased melanin synthesis and the inadequate transfer of the pigment from the melanocytes to the nearby keratinocytes. Increased melanin synthesis occurs with the tyrosinase enzyme's raised activity [11]. The increase in melanin synthesis is considered the leading mechanism in lieu of inadequate melanin transfer [6]. Electron microscopy showed the presence of extensive megaloblastic keratinocytes encircling the melanosomes containing melanocytes. The treatment consists of oral and/or parenteral vitamin B12 depending on the severity of symptoms and the level of deficiency. When hyperpigmentation is the primary presentation, oral treatment is preferred over parenteral treatment. The resolution period of the hyperpigmentation tends to vary from 6 to 12 weeks after the treatment [2]. Recurrence of hyperpigmentation in our patient was not observed at a one-year follow-up.

## Conclusions

A thorough systemic examination should be performed in patients presenting with hyperpigmentation of the skin. It should be investigated for a deficiency of vitamin B12, besides ruling out the other common causes. Alongside providing adequate treatment, the patient should be followed up for a few weeks to examine for the resolution and screen for neurological manifestations, as there can be a late manifestation in some patients.
